# Cultural Competence and Loneliness: Unveiling Hidden Connections Among Saudi Nurses

**DOI:** 10.3390/bs16050631

**Published:** 2026-04-23

**Authors:** Rasha Mohammed Hussien, Ghida Saleh Algeffari, Mahmoud Abdelwahab Khedr, Wafa Hamad Almegewly

**Affiliations:** 1 Department of Community, Psychiatric, and Mental Health Nursing, College of Nursing, Qassim University, Buraydah 52571, Saudi Arabia; rm.ahmed@qu.edu.sa; 2 Specialized Surgical Unit, Outpatient Department, Nursing Division, Qassim University, Medical City, Buraidah 51411, Saudi Arabia; ggeffari@qumc.edu.sa; 3 Psychiatric and Mental Health Nursing Department, Faculty of Nursing, Alexandria University, Alexandria 21526, Egypt; 4 Department of Community and Psychiatric Mental Health Nursing, College of Nursing, Princess Nourah bint Abdulrahman University, P.O. Box 84428, Riyadh 11671, Saudi Arabia

**Keywords:** culture, competence, loneliness, anxiety, depression, nurses

## Abstract

Background: Cultural competence is essential in nursing, enabling the delivery of ethical, patient-centered, and respectful care that respects diverse cultural backgrounds in an increasingly diverse healthcare setting. Improving cultural competence can substantially reduce stereotyping, time pressure, and distress among nurses. Objective: This study aimed to examine the relationship between cultural competence and loneliness among nurses working at a university medical city in Saudi Arabia and to identify associated demographic and psychological factors. Methods: A descriptive cross-sectional study was conducted using a convenience sample of 184 nurses. Data were collected via an online questionnaire that included the Cultural Capacity Scale, the Revised UCLA Loneliness Scale, and the Patient Health Questionnaire-4 between April and May 2024. Descriptive statistics, Spearman’s correlation, and multiple linear regression were used in the data analysis. Result: Findings indicate high cultural competence (mean score: 78.82) but moderate loneliness (mean score: 11.9). Notably, a strong negative correlation exists between cultural competence and feelings of loneliness (r = −0.777) and anxiety/depression (r = −0.818), suggesting that increased cultural competence is associated with lower loneliness and mental health issues. Conclusions: Both cultural knowledge and sensitivity emerged as significant predictors of lower anxiety and depression levels. These findings highlight the association between cultural competence and reduced loneliness and psychological distress among nurses, suggesting the need for targeted training interventions to improve nurses’ well-being and the quality of patient-centered care in culturally diverse healthcare settings.

## 1. Introduction

The mobility of the global nursing workforce is well documented in the nursing literature. The nursing workforce in Saudi Arabia comprises individuals from diverse ethnic backgrounds, creating a culturally diverse setting (Albougami et al., 2019). Such diversity requires nurses to possess higher cultural competence to address the disparities in health beliefs, communication, and care expectations (Almutairi et al., 2015a, 2015b).

Cultural competence is a critical component of nursing practice, enabling nurses to provide patient-centered care by assessing and distinguishing varied individuals’ healthcare beliefs, values, and practices (Zghal et al., 2021; Kaphle et al., 2022). It comprises four components: cultural awareness, cultural knowledge, cultural sensitivity, and cultural skills (Y. W. Liang et al., 2014; Brodowicz-Król et al., 2022). Several empirical investigations, particularly those in nursing research, have recognized this concept (Albougami & Alotaibi, 2020; Vandermause et al., 2021), and the learning is required for cultural competency rather than being an innate attribute (Ohta et al., 2022; H. Y. Liang et al., 2021).

The research findings indicated a need to enhance the cultural knowledge and skills of the nursing staff. Cultural awareness is the capacity to critically examine one’s professional and cultural beliefs to learn more about personal prejudices or understandings of other cultures. It also refers to the ability to recognize meaningfully that one’s cultural ideas differ from those of others (Y. W. Liang et al., 2014).

Cultural knowledge encompasses the capacity to acquire information pertaining to various cultural groups, including their health beliefs, health-seeking behaviors, perceptions of illness and morality, and the treatments they consider influential. Cultural sensitivity signifies the capacity to empathize with, trust, respect, and accept the beliefs and values of diverse populations while acknowledging the impact of a patient’s culture on their health-seeking behaviors. Cultural skills encompass the capacity to use tools and resources to enhance communication, acquire information about an individual’s cultural background, and perform a cultural assessment to address needs stemming from the patient’s background (Y. W. Liang et al., 2014; H. Y. Liang et al., 2021). 

Providing culturally sensitive treatment has several benefits. For instance, it improves prognosis by empowering patients, making them feel appreciated, and increasing compliance with treatment programs (Berie et al., 2021). By honoring each patient’s native cultural beliefs and practices and being sensitive to patients’ linguistic, cultural, lifestyle, and health-perception differences, NPs can improve healthcare quality, increase patient satisfaction, and eradicate healthcare inequity (Whitehead et al., 2022). However, neglecting these factors due to language barriers, miscommunication, or cultural insensitivity can impede the delivery of culturally appropriate healthcare services (H. Y. Liang et al., 2021). 

Research has shown that ineffective handling of cultural variety can result in dysfunctional behaviors (Klingler & Marckmann, 2016) and interpersonal disputes (Van Keer et al., 2015). According to Wesołowska et al. (2019), nurses in culturally diverse healthcare settings, whether native-born or foreign-born, may have less perceived time pressure at work, less discomfort, and better sleep quality if they practice cross-cultural empathy. According to another study, nurses with international training highly value acknowledgment and gratitude from patients, coworkers, and employers (Lee et al., 2021). 

Despite the growing body of evidence on cultural competence in nursing, the potential relationship between this competency and psychosocial outcomes, such as loneliness, remains underexplored. Although previous studies have examined cultural competence and stress among nurses, few have explored its association with loneliness, particularly in multicultural healthcare settings such as Saudi Arabia. Nurses with limited cultural competence may encounter challenges in communication and social integration, consequently heightening feelings of loneliness and social isolation. 

Loneliness is a subjective and personal experience characterized by feelings of detachment and separation from others (Willis et al., 2022). It can manifest in various domains, including personal relationships, workplaces, and community settings. In the healthcare sector, loneliness is prevalent and may arise from the demanding nature of the work, extended working hours, and the emotional stress associated with patient care (Lim et al., 2020). 

This study is guided by a stress-buffering conceptual framework, in which cultural competence is viewed as a protective resource that may reduce loneliness and psychological distress among nurses working in culturally diverse settings (Ellison et al., 2017). Cultural competence, including cultural awareness, knowledge, sensitivity, and skills, may improve interpersonal communication, strengthen social inclusion, and enhance nurses’ sense of belonging in the workplace. In turn, these processes may be associated with lower levels of loneliness, anxiety, and depression (Brodowicz-Król et al., 2022). The model also assumes that educational level, multicultural training, and prior exposure to diverse environments may strengthen cultural competence, while organizational context may influence the direction and magnitude of these relationships (Ellison et al., 2017). Accordingly, the present study hypothesizes that higher cultural competence is associated with lower loneliness and better mental health outcomes among nurses.

Experts have discovered that nurses deficient in cultural understanding and competency have elevated levels of perceived stress. Intercultural sensitivity has shown a substantial adverse correlation with nurses’ moderate levels of perceived stress (Uzun & Sevinç, 2015). The nurses’ worries over the care quality for patients from diverse ethnic backgrounds are evident (Markey et al., 2018). Patients’ needs, informed permission, and medicine were prioritized by nurses, who were most worried about their capacity to communicate with patients from diverse cultural backgrounds (Cruz et al., 2017).

The previous study suggested that healthcare professionals enhance their cultural sensitivity by engaging with individuals from diverse cultural backgrounds. It is essential to enhance language competence within the institutions and to establish plans that facilitate opportunities for physicians and nurses to acquire international experience and cultural sensitivity training (Aksoy & Akkoç, 2020). Moreover, improving psychosocial working conditions, especially by reducing negative factors in the work environment of high-strain and isolated high-strain jobs, should be considered to enhance cross-cultural competence among nursing staff (Wesołowska et al., 2019), thereby improving social integration, reducing workplace stress, and alleviating loneliness.

Nurses exhibit notably lower levels of cultural competence than other healthcare professionals (Casillas et al., 2014; Rakic et al., 2022). Effective educational training for nurses is suggested to enhance cultural competence and address health disparities (Osmancevic et al., 2023), reduce stereotyping, time pressure, and distress among nurses, delivering culturally competent care, empowering them to function effectively as team members and provide comprehensive patient-centered care (H. F. Liang et al., 2019). Therefore, this study explores the interplay between cultural competence and loneliness among hospital nurses at Medical City at Qassim University. Identifying the demographic and psychological factors related to these variables will yield important insights into the factors influencing the quality of patient-centered care in clinical environments.

### 1.1. Aim of the Study

This study aimed to examine the relationship between cultural competence and loneliness and to identify demographic and psychological predictors among nurses.

### 1.2. Research Questions

What is the relationship between cultural competence and loneliness among nurses?

Which demographic and psychological factors predict cultural competence and loneliness?

## 2. Methods

### 2.1. Study Design

The study espoused a descriptive research design using a cross-sectional method to evaluate the association level between the investigated variables. Due to the time-specific character of the data analysis, a cross-sectional design becomes operational.

### 2.2. Study Setting

Medical City at Qassim University provided services including outpatient clinics with different specialties, one-day surgical operations, endoscopy, and cardiac catheterization.

### 2.3. Sample Size

The sample size was calculated using the formula for a single population proportion with a 95% confidence level and a 5% margin of error, assuming a population proportion of 0.50 to maximize variability (Thompson, 2012). The total population of nurses in the study setting was 350. 

Using the parameters *N* = 350, *Z* = 1.96 (95% confidence level), *d* = 0.05 (margin of error), and *p* = 0.50, the required sample size was calculated as follows:

$$n=\frac{N\times Z^{2}\times p\left(1-p\right)}{d^{2}\left(N-1\right)+Z^{2}\times p\left(1-p\right)}\;n=\frac{350\times {\left(1.96\right)}^{2}\times \left(0.5\right)\left(0.5\right)}{{d\left(0.05\right)}^{2}\times \left(349\right)+{\left(1.96\right)}^{2}\times \left(0.5\right)\left(0.5\right)}\;n=\frac{350\times 0.9604}{0.8725+0.9604}\;n=\frac{336.14}{1.8329}\approx 183.4\approx 184$$

Therefore, the minimum required sample size was 184 participants.

*n* = Sample size, *N* = Total Population, *Z* = The standard value corresponding to a confidence level of 95%, which is (1.96), *d* = Margin of Error, here 0.05, and *p* = Population Proportion = 0.50.

#### Sampling Technique (With Inclusion and Exclusion Criteria)

A convenience sampling technique was used to recruit nurses who were willing to participate in the study and had at least 1 year of clinical experience. Those who chose not to participate in the study and those with less than 1 year of clinical experience were excluded.

### 2.4. Data Collection Methods

Part I—Sociodemographic characteristics: After reviewing the relevant literature, the researcher formulated this section to gather socio-demographic data of the study participants. The survey included questions about nurses’ age, marital status, educational background, work experience, prior multicultural education, and departmental affiliation.

Part II—The Cultural Capacity Scale (CCS): It comprises 20 items and was developed by Perng and Watson (2012). The CCS is a one-dimensional scale constructed by analyzing the Nurse Cultural Competence Scale (NCCS) using Mokken’s scaling method. The scale represents the respondents’ cultural knowledge, sensitivity, and skills. The items assess an individual’s competencies that can be developed in nursing care, including the articulation of cultural knowledge, application of communication skills, delivery of care, and instruction of others concerning culturally diverse patients. In response to the CCS, study participants will assess their level of agreement with the presented statements using a five-point Likert scale. A score of 1 indicates strongly disagree, while a score of 5 indicates strong agreement. The total attainable score is between 20 and 100. A higher score points to a greater level of cultural competence. The scale has demonstrated good validity and reliability. The Cronbach’s alpha of the CCS-A in this study was 0.9, as determined by Cruz et al. (2017). A prior study conducted in Saudi Arabia (Falatah et al., 2022) reported high scale reliability, as evidenced by a Cronbach’s alpha of 0.83. The Cronbach’s alpha score in the current study was 0.9, indicating high reliability.

**Part III:** The abbreviated version of the Revised UCLA Loneliness Scale (ULS-6) was adopted in the current study. It is one of the most popular measures for evaluating social isolation and loneliness (Russell et al., 2012). The preliminary scale had 20 assertions, but the ULS-6 had just six (Neto, 1992). The items were rated on a scale of four Likert points, where 1 represented ‘never’ and 4 denoted ‘frequently.’ A higher score indicated greater loneliness. The earlier research demonstrated a Cronbach’s standardized alpha of 0.703, signifying that the scale exhibited reliability (Hussien, 2022). The Cronbach’s alpha value of the current study was 0.76, signifying adequate reliability.

**Part IV—The Patient Health Questionnaire for Anxiety and Depression (PHQ-4 Score)**.

The Patient Health Questionnaire-4 (PHQ-4) was utilized to assess symptoms of depression and anxiety (Löwe et al., 2010). The two items of the PHQ-2 align with the symptoms outlined in the DSM-IV criteria for major depressive disorder (namely, anhedonia and depressive affect), while the two items of the GAD-2 correspond to the symptoms of generalized anxiety disorder (specifically, apprehension and anxiety). All four questions in this version use a 4-point Likert scale, with 0 meaning “not at all” and 3 meaning “nearly every day.” The time frame for the questions is within the previous two weeks. The cumulative score of the PHQ-4 (psychological distress) varies from 0 to 12, while the individual scores of its two subscales (PHQ-2 and GAD-2) vary from 0 to 6. The threshold for identifying likely instances of depression (PHQ-2) or anxiety (GAD-2) is 3 or higher for each subscale, while the criterion for probable psychological distress (PHQ-4) is 6 or higher for the overall scale (Cano-Vindel et al., 2018; Löwe et al., 2010; Mendoza et al., 2022). In recent research (Apputhurai et al., 2024), the PHQ-4 combined anxiety and depression subscales showed strong internal consistency, ranging from 0.78 to 0.89, while in the current study, the scale had a 0.856 internal consistency.

### 2.5. Pilot Study

After obtaining formal authorization from the relevant authorities, ten percent of the hospital nurses eligible for the study took part in a pilot study to assess the research instruments’ validity, reliability, transparency, and objectivity. Five mental health and psychiatric nursing specialists reviewed the instruments’ English versions, assessing them for cultural appropriateness, content validity, comprehensiveness, and clarity of items across various cultures. Pilot participants were excluded from the final analysis. It is considered that completing the questionnaire generally requires 20 to 30 min.

### 2.6. Data Collection Process

Data collection commenced following the acquisition of ethical and administrative approvals. Data gathering was conducted online via Google Forms. All utilized scales and demographic inquiries were incorporated into an online survey available in both English and Arabic. Participants were required to give consent by completing a consent form. An information sheet was provided detailing the study’s objectives, the voluntary nature of participation, and instructions for completing the questionnaire. The nursing office distributed the link to the staff nurses who satisfied the inclusion criteria. A reminder was issued one month later with the link resent. The survey was distributed to all eligible staff via official emails and WhatsApp groups. The survey is projected to take approximately 15 to 20 min to complete. Data collection commenced in April 2024 and concluded in May 2024.

### 2.7. Ethical Consideration

Permission was granted by the Committee of Research Ethics at Qassim University (24-83-02). Sending written permission from the hospital’s head to the medical city nursing department to get the staff to participate in the study was performed. The participants were apprised of their anonymity and voluntary participation, which was ensured by informed consent at the commencement of each online survey. Consent was obtained through two options: concur or disagree. The data would be distributed in an aggregated format for research purposes, as the participants were informed. Furthermore, the data was stored on a password-protected drive exclusively accessible to the research team.

### 2.8. Statistical Analysis

We used an IBM-compatible computer running Statistical Package for the Social Sciences (SPSS) version 25 for Windows to arrange, tabulate, and perform statistical analyses on the acquired data. The descriptive statistical methods were frequency, percentage, mean, and standard deviation. The Spearman correlation coefficient was used to assess any relationship between the variables under study. Multiple linear regression was used to examine predictors of loneliness, with cultural competence dimensions as independent variables. A level value was deemed significant when *p* < 0.05, while a level value was deemed very significant when *p* < 0.01. When *p* > 0.05, no statistically significant difference was taken into account.

## 3. Result

Table 1 shows that the majority of the studied nurses (63.6%) were between 30 and 40 years old, with a mean age of 33.85 ± 6.09 years. Most were female (85.3%) and married (60.9%). In terms of education, 73.4% held bachelor’s degrees and 24.5% had master’s or PhD degrees. Over one third were Filipino (37.5%), followed by Indian (34.2%) and Egyptian (11.4%) nurses. Regarding work experience, 38.6% had 6–10 years as nursing professionals, with a mean of 11.2 ± 3.35 years. Nurses were distributed across medical/surgical wards (33.2%), critical care units (32.6%), and the emergency department (31.0%). A significant proportion (37.5%) reported good proficiency in foreign languages, while 28.3% and 31.0% rated their proficiency as excellent and very good, respectively. However, the majority (64.1%) had not received any education or workshops on multicultural nursing. In terms of self-perceived cultural competence, 41.3% felt somewhat competent, 25.0% very competent, while 15.8% and 4.3% felt somewhat and very incompetent, respectively.

Table 2 indicates that nurses scored highest in cultural skills (Mean = 47.80 ± 10.6, 79.7%), followed closely by cultural sensitivity (Mean = 7.96 ± 1.84, 79.6%), while cultural knowledge received a slightly lower mean score (Mean = 23.05 ± 5.49, 76.8%). The overall culture competency score is 78.82 ± 17.3, reflecting a strong level of cultural competency among participants, with an average of 78.2%. It also presents that the total loneliness score is 11.9 ± 3.61, with the highest mean score in the “feel part of a group of friends” (Mean = 3.10 ± 0.99) and lack companionship (Mean = 2.01 ± 0.96), indicating a moderate level of loneliness among the participants. Overall, the findings suggested that while nurses generally experience social connectedness, some degree of emotional disconnection may persist despite physical presence.

**Figure 1:**
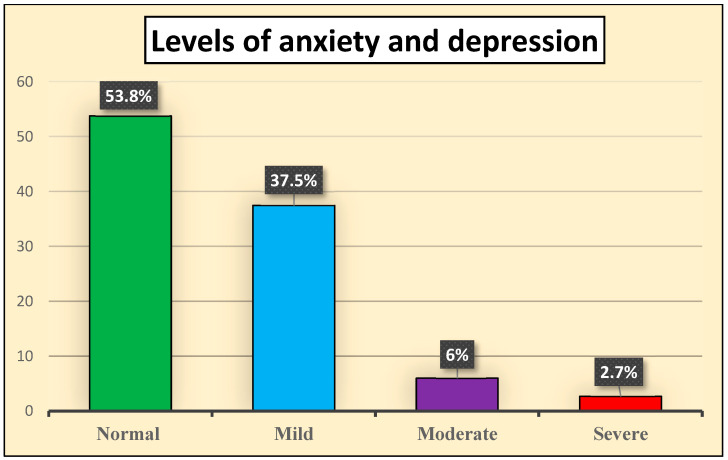
Percentage distribution of the studied nurses according to their levels of anxiety and depression (*n* = 184).

Figure 1 illustrates the percentage distribution of anxiety levels among the studied nurses. The data shows that more than half of the nurses, specifically 53.8%, reported having a normal level of anxiety. Meanwhile, 37.5% exhibited mild anxiety symptoms, indicating that a significant portion of this group experiences some level of anxiety. Additionally, 6% of the nurses were categorized as having moderate anxiety, while only 2.7% reported severe anxiety levels.

Table 3 shows that education level, participation in multicultural nursing workshops, and self-assessed cultural competence are strongly positively correlated with cultural skills, knowledge, sensitivity, and overall cultural competency (r > 0.5, *p* < 0.001). Conversely, nationality exhibits a weak negative correlation with the dimensions of cultural competency (r < −0.2, *p* < 0.05). Notably, education level, attendance at multicultural workshops, and self-perceived cultural competence also demonstrate strong negative correlations with loneliness and anxiety/depression (r < −0.5, *p* < 0.001).

Table 4 indicates strong positive correlations among the dimensions of cultural competency, with cultural knowledge (r = 0.887, *p* < 0.001) and cultural sensitivity (r = 0.825, *p* < 0.001) exhibiting particularly high associations with cultural skills. Additionally, the total cultural competency score is significantly correlated with loneliness (r = −0.777, *p* < 0.001) and total anxiety and depression (r = −0.818, *p* < 0.001), suggesting that greater cultural competency is linked to lower levels of loneliness and mental health issues. On the other hand, there is a positive correlation between loneliness and anxiety/depression (r = 0.785, *p* < 0.001), indicating that as feelings of loneliness increase, so do levels of anxiety and depression.

Table 5 details the results from multiple linear regression analyses that explore the relationships between cultural competency and loneliness among a cohort of 184 nurses. In Model 1, the overall cultural competency score shows a significant negative correlation with loneliness (B = −0.161, *p* < 0.001), suggesting that increased cultural competency is associated with reduced feelings of loneliness. Model 2 further investigates this relationship by analyzing three specific dimensions of cultural competency: cultural skills, cultural knowledge, and cultural sensitivity. Each dimension reveals a significant negative relationship with loneliness, with cultural sensitivity exhibiting the most substantial effect (B = −1.389, *p* < 0.001), followed by cultural knowledge (B = −0.509, *p* < 0.001) and cultural skills (B = −0.254, *p* < 0.001). The R^2^ values indicate that these models account for a significant portion of the variance in loneliness, with Model 2 explaining 60.1% of the variance. In Model 3, when all dimensions are analyzed together, cultural knowledge (B = −0.316, *p* < 0.001) and cultural sensitivity (B = −0.365, *p* = 0.030) continue to be significant predictors of loneliness, while cultural skills do not show a significant effect (*p* = 0.190).

Table 6 presents the findings from multiple linear regression analyses that explore the relationship between cultural competency and anxiety and depression among 184 nurses. In Model 1, the overall cultural competency score is significantly linked to anxiety and depression (B = −0.125, *p* < 0.001), indicating that greater cultural competency is associated with lower levels of anxiety and depression. Model 2 breaks this down into three dimensions of cultural competency: cultural skills, cultural knowledge, and cultural sensitivity. Each dimension shows a significant negative association with anxiety and depression, with cultural knowledge (B = −0.392, *p* < 0.001) and cultural sensitivity (B = −1.107, *p* < 0.001) having the most substantial impacts. The R^2^ values suggest that these models account for a significant amount of variance in anxiety and depression, with Model 2 explaining 65.5% of the variance. In Model 3, where all dimensions are analyzed together, cultural knowledge (B = −0.209, *p* < 0.001) and cultural sensitivity (B = −0.402, *p* < 0.001) remain significant predictors, while cultural skills do not show a significant effect (*p* = 0.126).

Table 7 reveals a significant moderation effect (interaction B = −0.215, *p* = 0.005), confirming that cultural competence acts as a protective buffer that mitigates the impact of work environment stressors on nurse anxiety. Furthermore, the mediation analysis identified a significant indirect effect (B = −0.323, Bootstrap 95% CI [−0.41, −0.23]), suggesting that cultural competence serves as a primary psychological mechanism through which the work environment influences mental health outcomes.

## 4. Discussion

As the nursing workforce increasingly interacts with patients from various cultural backgrounds, understanding how cultural competence influences emotional well-being becomes essential. This research seeks to identify specific dimensions of cultural competence—such as cultural knowledge, skills, and sensitivity—that may mitigate feelings of loneliness and enhance social connections among nurses.

The high scores in cultural skills among nurses indicate their proficiency in applying their understanding of cultural differences in practical care scenarios. The high level of cultural competence observed in this study may be significantly influenced by the internal policies and institutional characteristics of Qassim University Medical City. As a leading academic medical center, the institution adheres to rigorous national (CBAHI) and international (JCI) accreditation standards, which explicitly mandate training in patient-centered, culturally appropriate care. Furthermore, the institutional policy of maintaining a highly diverse workforce—comprising over 85% expatriate nurses from various countries (Table 1)—creates an environment of ‘cultural immersion.’

This proficiency is essential for effective interpersonal and intercultural communication, which fosters cultural sensitivity among healthcare providers. Nurses with strong cultural skills are better equipped to navigate barriers such as language differences and non-verbal cues, crucial for building rapport with patients from diverse backgrounds (Kwame & Petrucka, 2021). This ability not only enhances the quality of care but also promotes patient trust and satisfaction. These observations are in line with studies suggesting that communication competence is a key component of culturally responsive nursing practice (Kwame & Petrucka, 2021; Ličen & Prosen, 2023).

Cultural sensitivity scores closely follow those of cultural skills, suggesting that nurses recognize and appreciate the differences among their patients, which is vital for providing individualized care that respects cultural beliefs and practices. Research indicates that culturally sensitive care can lead to improved health outcomes and greater patient satisfaction (Tucker et al., 2011). This aligns with prior evidence reporting that empathy and respect for cultural diversity are often more closely tied to positive patient-related outcomes than technical knowledge alone (Rukadikar et al., 2022).

While cultural knowledge scores are somewhat lower, they still provide a solid foundation for nurses. Cultural knowledge involves understanding the health beliefs, practices, and values of diverse patient populations; insufficient knowledge in this area can hinder appropriate care and lead to misunderstandings regarding patients’ needs (Liu et al., 2022). By developing a deeper understanding of various cultures, nurses can enhance their ability to deliver culturally competent care, ultimately improving health outcomes for patients.

Moreover, the overall score indicates a moderate level of loneliness among the participants, reflecting a nuanced interplay of social connections and feelings of isolation within the nursing profession. This moderate loneliness suggests that while nurses may not experience extreme isolation, they face significant emotional challenges that need to be addressed (Hawkins et al., 2023). Loneliness among healthcare professionals can profoundly impact mental health, job satisfaction, and overall performance, particularly in high-stress environments characterized by demanding workloads (Søvold et al., 2021). Comparable studies in healthcare settings have similarly shown that loneliness may persist even when staff report functional teamwork, suggesting that physical proximity does not necessarily translate into emotional connectedness (Stubbs & Achat, 2022; Dyrbye et al., 2017).

Interestingly, the item “Feel isolated from others” received one of the lowest scores, indicating that nurses do not primarily perceive themselves as isolated. This may imply that although colleagues physically surround them, they lack emotional connections, which aligns with research showing that emotional isolation can occur even in crowded settings (Hammoud et al., 2021). Conversely, the item “Feel part of a group of friends” scored higher than other loneliness indicators, suggesting that nurses do experience some sense of belonging within their social circles, which is crucial for mitigating loneliness and enhancing job satisfaction (Stubbs & Achat, 2022). However, higher scores on items like “Lack companionship” and “People are around me but not with me” highlight feelings of emotional distance despite physical proximity to others. This disconnection can lead to increased stress and burnout among nurses, emphasizing the need for fostering deeper interpersonal connections within nursing teams (Kinman et al., 2023).

The current study findings indicate that education level, participation in multicultural nursing workshops, and self-assessed cultural competence are strongly positively correlated with cultural skills, knowledge, sensitivity, and overall cultural competency among nurses. This suggests that higher educational attainment and active involvement in multicultural training significantly enhance nurses’ abilities to deliver culturally competent care. Previous research supports this notion, demonstrating that educational interventions focused on cultural competence can effectively improve healthcare providers’ understanding and skills in working with diverse populations (Echeverri & Chen, 2016; Liu et al., 2022). Additionally, participation in workshops promotes experiential learning, which is essential for developing the competencies needed to navigate complex cultural interactions (Walkowska et al., 2023). Conversely, nationality shows a weak negative correlation with cultural competency dimensions, which suggests that nationality alone is not a strong determinant of cultural competence and should be interpreted cautiously. This finding indicates that any observed differences between national and expatriate nurses may be influenced more by education, prior multicultural exposure, and training opportunities than by nationality itself. Therefore, a dedicated subgroup comparison would be needed to determine whether there is a meaningful difference in competence between Saudi and non-Saudi nurses. Addressing these disparities through tailored educational programs could help bridge the gap and foster inclusivity in healthcare settings (Latif, 2020).

A significant portion of nurses reported normal anxiety levels, indicating that many are managing their stress effectively and are likely able to perform their duties with competence and confidence. However, the presence of a notable percentage experiencing mild anxiety raises important concerns, as even mild symptoms can impact job performance and overall well-being over time (Roberts et al., 2021). This suggests a potential risk for increased stress and burnout if not addressed adequately. Furthermore, the existence of nurses with moderate and severe anxiety highlights the need for immediate attention, as these individuals may struggle with their responsibilities and face challenges that could affect patient care and safety.

The study reveals strong negative correlations between education level, attendance at multicultural workshops, self-perceived cultural competence, and loneliness, indicating that cultural competence serves as a protective factor against feelings of isolation among nurses. Nurses who engage in continuous education and self-assessment of their cultural skills tend to experience lower levels of loneliness, which aligns with existing literature suggesting that enhanced cultural competence fosters stronger social connections and support systems among healthcare professionals (Echeverri & Chen, 2016). This is similar to findings from earlier studies in culturally diverse healthcare environments, where confidence in cross-cultural communication was associated with stronger workplace belonging and reduced social disconnection (Chen, 2019; Dyrbye et al., 2017).

Additionally, the negative correlation with anxiety and depression highlights the importance of cultural competence in buffering mental health challenges; nurses who feel competent in understanding and interacting with diverse patient populations may experience reduced stress associated with cross-cultural interactions (Kaihlanen et al., 2019). This is particularly relevant in high-pressure hospital environments where emotional resilience is essential for maintaining personal well-being and professional effectiveness. 

The positive correlations among the dimensions of cultural competency emphasize their interdependence in shaping nurses’ abilities to provide culturally sensitive care. Cultural knowledge and sensitivity are particularly associated with cultural skills, suggesting that a solid understanding of cultural differences and empathy are crucial for developing practical skills in cross-cultural interactions. This supports the need for nursing education programs to integrate theoretical knowledge with practical skills in cultural competence training professionals (Rukadikar et al., 2022). This pattern mirrors previous research indicating that the components of cultural competence are mutually reinforcing rather than independent constructs (Chen, 2019; Dyrbye et al., 2017).

The significant negative correlations between overall cultural competency scores and feelings of loneliness, anxiety, and depression further underline the protective role of cultural competence in promoting mental health and well-being among nurses. From a stress-buffering perspective, cultural competence may function as a critical psychosocial resource that reduces feelings of isolation through several interconnected mechanisms (Ellison et al., 2017). As nurses enhance their cultural competence, they may experience lower levels of loneliness and reduced symptoms of anxiety and depression, leading to improved job satisfaction and resilience against workplace stressors (Dyrbye et al., 2017). Conversely, the positive correlation between loneliness and anxiety/depression indicates that increased feelings of isolation can exacerbate psychological distress, raising concerns about the potential for burnout in the nursing profession (Rukadikar et al., 2022).

The multiple linear regression analyses reveal significant insights into the relationship between cultural competency and feelings of loneliness among nurses. The findings indicate a strong negative correlation between overall cultural competency and loneliness, suggesting that higher levels of cultural competency are associated with lower feelings of loneliness. This aligns with existing research that identifies cultural competence as a protective factor against loneliness in healthcare professionals, as it enhances their ability to navigate cross-cultural interactions and fosters social connections in the workplace (Dyrbye et al., 2017). However, given the high correlations among the cultural competence subscales, multicollinearity may have affected the stability of the regression estimates, particularly in models including all dimensions simultaneously. Future studies should examine variance inflation factors, tolerance values, or alternative dimension reduction approaches to clarify the unique contribution of each component.

When examining specific dimensions of cultural competency, such as cultural skills, knowledge, and sensitivity, each aspect demonstrates a significant negative relationship with loneliness. Notably, cultural sensitivity emerges as the most influential factor, highlighting that nurses who can empathize with diverse patients are better equipped to form meaningful connections, thereby mitigating feelings of isolation. Further analysis indicates that while cultural knowledge and sensitivity are significant predictors of loneliness, cultural skills do not show a significant effect when considered alongside the other dimensions. This suggests that understanding cultural differences and empathizing with diverse perspectives are particularly crucial for reducing loneliness in nursing, reinforcing the importance of effective cross-cultural communication and trust-building in healthcare settings. Overall, these findings underscore the necessity of fostering cultural competence within nursing to enhance both nurse well-being and patient care quality (Dyrbye et al., 2017; Stubbs & Achat, 2022).

The analysis of cultural competency among nurses reveals a significant negative correlation between overall cultural competency and levels of anxiety and depression, indicating that as nurses enhance their cultural competency, they experience reduced psychological distress. This finding supports existing literature that emphasizes the protective effects of cultural competence on mental health in healthcare providers (Dyrbye et al., 2017). The examination of specific dimensions—cultural skills, knowledge, and sensitivity—demonstrates that cultural knowledge and sensitivity are particularly influential in mitigating anxiety and depression, while cultural skills show less impact.

The integrated mediation and moderation analysis provides a robust expansion of our understanding of the relationship between cultural competence and psychological distress. A key finding is the significant interaction effect, which confirms that cultural competence functions as a protective moderator (buffer) within the nursing work environment. This denotes that while environmental stressors may typically elevate anxiety, nurses with higher levels of cultural competence are less susceptible to these negative impacts. Furthermore, the significant indirect effect establishes cultural competence as a critical mediating mechanism. Nevertheless, these mediation and moderation findings should also be viewed cautiously in light of the cross-sectional design, which limits temporal ordering and causal interpretation. This indicates that the work environment’s influence on anxiety is partially explained by its impact on a nurse’s perceived cultural capacity (Kiptulon et al., 2024). A longitudinal design would be better suited to test the proposed directionality and mediation pathway.

## 5. Conclusions

The findings from this study underscore the critical importance of cultural competency in nursing, revealing significant associations between cultural competence and both mental health outcomes and feelings of loneliness among nurses. Higher overall cultural competency is associated with lower levels of loneliness, anxiety, and depression, highlighting that nurses who are well-versed in cultural skills, knowledge, and sensitivity tend to report better mental well-being. Specifically, cultural sensitivity and cultural knowledge emerged as key predictors of lower anxiety and depression levels, suggesting that targeted educational interventions in these areas may be valuable for enhancing nurses’ ability to connect with diverse patient populations while also supporting their own mental health. These findings support the integration of cultural competence training into nursing professional development programs and workplace policies. In particular, structured and ongoing intercultural training may help strengthen cross-cultural communication, promote inclusion, and reduce feelings of social isolation among nursing staff. Future longitudinal studies are needed to examine causal relationships and to evaluate the effectiveness of such interventions over time.

## 6. Study Limitations

This study has several limitations that should be acknowledged. Firstly, the cross-sectional design restricts the ability to establish causal relationships between cultural competence, loneliness, and mental health outcomes among nurses. Consequently, it is difficult to determine whether increased cultural competence is associated with reduced loneliness or if less loneliness fosters greater cultural awareness. Therefore, the findings should be interpreted as associations rather than evidence of causation, even though some correlations were strong. Secondly, the reliance on self-reported measures may introduce bias, as participants may overestimate their cultural competence or underreport feelings of loneliness and anxiety due to social desirability. This concern is particularly relevant for culturally competence-related items, where respondents may have provided socially acceptable answers rather than fully accurate self-assessments. Additionally, the study’s sample was drawn from a single institution, which may limit the generalizability of the findings to other healthcare settings or regions. The use of an online questionnaire distributed through email and WhatsApp may also have introduced selection bias, as it may have favored nurses who were more engaged with digital communication platforms. Lastly, the potential influence of unmeasured confounding variables, such as personal stressors or organizational culture, may also impact the results. Accordingly, the strong correlations observed in this study should be viewed cautiously, as they reflect relationships within a specific sample and do not establish direct or causal effects.

## 7. Recommendations

To address these limitations, future research should consider longitudinal designs that can better assess causal relationships between cultural competence and mental health outcomes. Expanding the sample size to include multiple healthcare institutions could enhance the generalizability of the findings. Furthermore, incorporating qualitative methods, such as interviews or focus groups, would provide deeper insights into nurses’ lived experiences of cultural competence and loneliness. Also, to explore nurses’ subjective experiences, perceptions, and the contextual factors influencing loneliness, which provide valuable insights into the causes to develop targeted interventions. Educational interventions should be developed and evaluated to enhance cultural competence among nursing staff, focusing on cultural sensitivity and knowledge. Regular workshops and training sessions could foster ongoing professional development and help nurses effectively engage with diverse patient populations. In addition, hospitals should implement structured intercultural training programs as part of orientation and continuing education to strengthen nurses’ cross-cultural communication skills. Clinical managers should also consider interventions aimed at reducing social isolation among professionals, such as peer-support groups, team-building activities, and mentorship programs that promote inclusion and workplace belonging. At the policy level, integrating cultural competence into competency frameworks, appraisal systems, and staff development plans may help ensure that cultural responsiveness becomes a sustained organizational priority rather than a one-time educational activity. These actions could improve both nurse well-being and the quality of patient-centered care in culturally diverse settings.

## 8. Clinical Implications for Nursing Practice

The findings of this study have significant implications for clinical practice in nursing. Enhancing cultural competence among nurses is crucial for delivering patient-centered care in increasingly diverse healthcare settings. The results indicate that nurses with higher cultural competence experience lower levels of loneliness, anxiety, and depression, which can directly impact their job performance and overall well-being. By prioritizing cultural competence training, healthcare organizations can create supportive environments that not only improve nurses’ mental health but also enhance patient satisfaction and health outcomes. Furthermore, fostering a culture of empathy and understanding within nursing teams can strengthen interpersonal relationships, thereby reducing feelings of isolation and promoting collaboration in patient care. Overall, integrating cultural competence into nursing practice can lead to a more effective and compassionate healthcare delivery system.

## Figures and Tables

**Table 1:** Frequency distribution of the studied nurses according to their socio-demographic characteristics (*n* = 184).

Socio-Demographic Characteristics of the Studied Nurses	No.	%
**Age (Years)**		
20–<30	48	26.1
30–<40	117	63.6
40–<50	17	9.2
≥50	2	1.1
**Range**	**(23–51)**	
**Mean ± SD**	**33.85 ± 6.09**	
**Gender**		
Male	27	14.7
Female	157	85.3
**Marital status**		
Single	63	34.2
Married	112	60.9
Divorced/separated	7	3.8
Widowed	2	1.1
**Educational level**		
Diploma	4	2.2
Bachelor degree	135	73.4
Master’s or PhD	45	24.5
**Nationality**		
Saudi	23	12.5
Egyptian	21	11.4
Jordanian	5	2.7
India	63	34.2
Filipino	69	37.5
Others	3	1.6
**Work experience as a nursing professional (Years)**		
1–5	44	23.9
6–10	71	38.6
11–15	45	24.5
≥16	24	13.0
**Range**	**(2–26)**	
**Mean ± SD**	**11.2 ± 3.35**	
**Department**		
Emergency	57	31.0
Critical care units	60	32.6
Medical/Surgical wards	61	33.2
Outpatient clinics	6	3.3
**Foreign language proficiency level**		
Excellent	52	28.3
Very good	57	31.0
Good	69	37.5
Poor	6	3.3
**Education (workshop) on multicultural nursing**		
Yes	66	35.9
No	118	64.1
**Self-perceived cultural competence**		
Very incompetent	8	4.3
Somewhat incompetent	29	15.8
Neither competent nor incompetent	25	13.6
Somewhat competent	76	41.3
Very competent	46	25.0

SD = Standard deviation.

**Table 2:** Mean score of the culture competency and loneliness as reported by the studied nurses (*n* = 184).

Culture Competency	Min	Max	Mean ± SD	Mean %	Rank
**Cultural skills**	24	60	47.80 ± 10.6	79.7%	1
**Cultural knowledge**	12	30	23.05 ± 5.49	76.8%	3
**Cultural sensitivity**	3	10	7.96 ± 1.84	79.6%	2
**Total culture competency score**	**39**	**100**	78.82 ± 17.3	**78.2%**	
**Loneliness**	**Min**	**Max**	**Mean ± SD**		
**Lack companionship**	1	4	2.01 ± 0.96		
**Feel part of a group of friends**	1	4	3.10 ± 0.99		
**Feel left out**	1	4	1.72 ± 0.84		
**Feel isolated from others**	1	4	1.63 ± 0.76		
**Feel unhappy and withdrawn**	1	4	1.71 ± 0.88		
**People are around me, but not with me.**	1	4	1.74 ± 0.87		
**Total loneliness score**	**6**	**21**	**11.9 ± 3.61**		

SD = Standard deviation.

**Table 3:** Correlation between nurses’ demographic data and total culture competency dimensions, total loneliness, total anxiety, and depression (*n* = 184).

Variables		Cultural Skills	Cultural Knowledge	Cultural Sensitivity	Total Culture Competency Score	Loneliness	Total Anxiety and Depression
**Age**	**r** ***p***	−0.053 0.475	−0.022 0.762	−0.052 0.484	−0.050 0.503	0.050 0.503	−0.024 0.751
**Gender**	**r** ***p***	0.112 0.131	0.042 0.568	0.136 0.065	0.083 0.263	−0.067 0.363	−0.063 0.211
**Marital status**	**r** ***p***	−0.041 0.577	−0.062 0.400	0.009 0.904	−0.043 - 0.562	0.013 0.857	0.070 0.342
**Education level**	**r** ***p***	0.627 0.000 **	0.597 0.000 **	0.544 0.000 **	0.649 0.000 **	−0.511 0.000 **	−0.552 0.000 **
**Nationality**	**r** ***p***	−0.259 0.000 **	−0.162 0.028 *	−0.178 0.016 *	−0.220 0.003 **	0.261 0.000 **	0.066 0.374
**Department**	**r** ***p***	−0.047 0.528	−0.027 0.717	−0.052 0.486	−0.044 0.552	0.049 0.513	−0.045 0.544
**Foreign language proficiency level**	**r** ***p***	0.041 0.584	0.034 0.644	0.089 0.229	0.031 0.673	0.082 0.266	−0.056 0.447
**Attendance workshop on multicultural nursing**	**r** ***p***	0.759 0.000 **	0.770 0.000 **	0.676 0.000 **	0.802 0.000 **	−0.693 0.000 **	−0.714 0.000 **
**Self-perceived cultural competence**	**r** ***p***	0.671 0.000 *	0.637 0.000 **	0.583 0.000 **	0.670 0.000 **	−0.524 0.000 **	−0.572 0.000 **

r = Correlation coefficient test. (-) = Negative correlation. *p* = *p*-value. No significant at *p* > 0.05. * *p* ≤ 0.05. ** *p* ≤ 0.001. Interpretation of r: Weak (0.1–0.24), Intermediate (0.25–0.74), Strong (0.75–0.99), and Perfect (1).

**Table 4:** Correlation between total culture competency dimensions, total loneliness, and total anxiety and depression among the studied nurses (*n* = 184).

Variables		Cultural Skills	Cultural Knowledge	Cultural Sensitivity	Total Culture Competency Score	Loneliness
**Cultural knowledge**	**r** ***p***	0.887 0.000 **	1			
**Cultural sensitivity**	**r** ***p***	0.825 0.000 **	0.825 0.000 **	1		
**Total culture competency score**	**r** ***p***	0.978 0.000 **	0.946 0.000 **	0.857 0.000 **	1	
**Loneliness**	**r** ***p***	−0.746 0.000 **	−0.775 0.000 **	−0.709 0.000 **	−0.777 0.000 **	1
**Anxiety and depression**	**r** ***p***	−0.786 0.000 **	−0.809 0.000 **	−0.768 0.000 **	−0.818 - 0.000 **	0.785 0.000 **

r = Correlation coefficient test. (-) = Negative correlation. *p* = *p*-value. No significant at *p* > 0.05. ** *p* ≤ 0.001. Interpretation of r: Weak (0.1–0.24), Intermediate (0.25–0.74), Strong (0.75–0.99), and Perfect (1).

**Table 5:** Multiple linear regression examining associations of culture competency with loneliness among the studied nurses (*n* = 184).

Items	B	Beta	95% Confidence Interval		t	*p* Value	R^2^	ANOVA	
			Lower	Upper				F	*p* Value
**Model 1**							0.603	276.92	0.000 **
**Overall culture competency**	−0.161	−0.777	−0.181	−0.142	16.64	0.000 **			
**Model 2**									
**Cultural skills**	−0.254	−0.746	−0.287	−0.220	15.12	0.000 **	0.557	228.81	0.000 **
**Cultural knowledge**	−0.509	−0.775	−0.570	−0.449	16.54	0.000 **	0.601	273.6	0.000 **
**Cultural sensitivity**	−1.389	−0.709	−1.590	−1.187	13.57	0.000 **	0.503	184.30	0.000 **
**Model 3**							0.620	97.97	0.000 **
**Cultural skills**	−0.053	−0.155	−0.131	0.026	1.317	0.190			
**Cultural knowledge**	−0.316	−0.480	−0.468	−0.164	4.099	0.000 **			
**Cultural sensitivity**	−0.365	−0.187	−0.694	−0.036	2.190	0.030 *			

Dependent Variable: Loneliness. Model 2: Each dimension of culture competency in a separate model. Model 3: All dimensions of culture competency in the same model. B = Unstandardized Coefficients. Beta = Standardized Coefficients. t: Independent *t*-test. R^2^ = Coefficient of multiple determination. No significant at *p* > 0.05. * *p* ≤ 0.05. ** *p* ≤ 0.001.

**Table 6:** Multiple linear regression examining associations of culture competency with anxiety and depression among the studied nurses (*n* = 184).

Items	B	Beta	95% Confidence Interval		t	*p* Value	R^2^	ANOVA	
			Lower	Upper				F	*p* Value
**Model 1**							0.669	368.27	0.000 **
**Overall culture competency**	−0.125	−0.818	−0.138	−0.112	19.19	0.000 **			
**Model 2**									
**Cultural skills**	−0.197	−0.786	−0.219	−0.174	17.14	0.000 **	0.618	293.91	0.000 **
**Cultural knowledge**	−0.392	−0.809	−0.433	−0.350	18.58	0.000 **	0.655	345.55	0.000 **
**Cultural sensitivity**	−1.107	−0.768	−1.242	−0.972	16.17	0.000 **	0.590	261.69	0.000 **
**Model 3**							0.691	134.42	0.000 **
**Cultural skills**	−0.041	−0.163	−0.093	0.012	1.539	0.126			
**Cultural knowledge**	−0.209	−0.431	−0.310	−0.108	4.084	0.000 **			
**Cultural sensitivity**	−0.402	−0.279	−0.620	−0.183	3.629	0.000 **			

Dependent Variable: Anxiety and depression. Model 2: Each dimension of culture competency in a separate model. Model 3: All dimensions of culture competency in the same model. B = Unstandardized Coefficients. Beta = Standardized Coefficients. t: Independent *t*-test. R^2^ = Coefficient of multiple determination. No significant at *p* > 0.05. ** *p* ≤ 0.001.

**Table 7:** Integrated Mediation and Moderation Analysis of Cultural Competence on the Relationship between Work Environment and Anxiety (*n* = 184).

Analysis Type	Variable Path/Term	Unstandardized B	SE	t/z	*p*-Value	95% CI [LL, UL]
**A. Moderation (Buffering)**	(Constant)	4.125	0.312	13.22	<0.001	[3.51, 4.74]
	Work Environment (X)	0.142	0.084	1.69	0.092	[−0.02, 0.31]
	Cultural Competence (W)	−0.684	0.052	−13.15	<0.001	[−0.79, −0.58]
	Interaction (X\times W)	−0.215	0.076	−2.83	0.005 *	[−0.36, −0.07]
**B. Mediation (Mechanism)**	Total Effect (c)	0.485	0.055	8.81	<0.001	[0.38, 0.59]
	Direct Effect (c’)	0.162	0.071	2.28	0.024 *	[0.02, 0.30]
	Indirect Effect (a\times b)	−0.323	0.045	—	<0.001 **	[−0.41, −0.23]
**Model Statistics**	R^2^ = 0.688	F = 132.1		*p* < 0.001		

Note: SE = Standard Error; CI = Confidence Interval; LL = Lower Limit; UL = Upper Limit. Part A (Moderation) tests the protective interaction term (X\times W). Part B (Mediation) utilizes 5000 bootstrap samples to test the indirect pathway. * *p* ≤ 0.05. ** Significant at *p* < 0.001.

## Data Availability

Data underpinning the findings of this study can be obtained from the corresponding author upon a reasonable request.
